# Formation of a Purple Product upon the Reaction of ABTS Radicals with Proteins

**DOI:** 10.3390/ijms24108912

**Published:** 2023-05-17

**Authors:** Kacper Kut, Ireneusz Stefaniuk, Grzegorz Bartosz, Izabela Sadowska-Bartosz

**Affiliations:** 1 Laboratory of Analytical Biochemistry, Institute of Food Technology and Nutrition, College of Natural Sciences, University of Rzeszow, 4 Zelwerowicza Street, 35-601 Rzeszow, Poland; kkut@ur.edu.pl; 2 Institute of Materials Engineering, College of Natural Sciences, University of Rzeszow, 1 Pigonia Street, 35-310 Rzeszow, Poland; istefaniuk@ur.edu.pl; 3 Department of Bioenergetics, Food Analysis and Microbiology, Institute of Food Technology and Nutrition, College of Natural Sciences, University of Rzeszow, 4 Zelwerowicza Street, 35-601 Rzeszow, Poland; gbartosz@ur.edu.pl

**Keywords:** BSA, tyrosyl radical, tyrosine adduct, ABTS, free radical, protein

## Abstract

The reaction of the 2,2′-azino-bis(3-ethylbenzothiazoline-6-sulfonate) free radical (ABTS^●^) with proteins (bovine serum albumin, blood plasma, egg white, erythrocyte membranes, and Bacto Peptone) leads not only to a reduction of ABTS^●^ but also to the appearance of a purple color (absorption maximum at 550–560 nm). The aim of this study was to characterize the formation and explain the nature of the product responsible for the appearance of this color. The purple color co-precipitated with protein, and was diminished by reducing agents. A similar color was generated by tyrosine upon reaction with ABTS^●^. The most feasible explanation for the color formation is the addiction of ABTS^●^ to proteins’ tyrosine residues. The product formation was decreased by nitration of the bovine serum albumin (BSA) tyrosine residues. The formation of the purple product of tyrosine was optimal at pH 6.5. A decrease in pH induced a bathochromic shift of the spectra of the product. The product was not a free radical, as demonstrated by electrom paramagnetic resonance (EPR) spectroscopy. Another byproduct of the reaction of ABTS^●^ with tyrosine and proteins was dityrosine. These byproducts can contribute to the non-stoichiometry of the antioxidant assays with ABTS^●^. The formation of the purple ABTS adduct may be a useful index of radical addition reactions of protein tyrosine residues.

## 1. Introduction

The assay of decolorization of the 2,2′-azino-bis (3-ethylbenzthiazoline-6-sulfonate) radical (ABTS^●^ decolorization assay) is one of the most popular methods of estimating the antioxidant capacity of food, plant extracts, and biological fluids, as well as the antioxidant activity of individual compounds [1,2,3,4,5]. This assay is based on one-electron reactions of reduction of the ABTS radical (ABTS^●^) by antioxidant compounds present in a sample. The assay is performed under conditions of ABTS^●^ excess, so a fraction of ABTS^●^ always remains after the measurement, enabling a precise measurement of the amount of ABTS^●^ reduced by a known amount of the tested material. The tacit assumption of the assay is that the decolorization of ABTS^●^ is entirely due to its reduction by antioxidants, and thus quantitatively reflects the amount of antioxidants present in a sample [1,2,6,7]. However, the reactions of the ABTS^●^ radical may be more complex even in this simple assay, and various concurrent reaction pathways may complicate its stoichiometry [8].

Incidentally, when measuring the antioxidant capacity of protein samples, we noticed the appearance of a purple color in samples left after the assay. This color formation must be a reflection of a reaction of the ABTS^●^ radical, normally escaping detection during the assay due to the presence of residual ABTS^●^. As the decolorization of ABTS^●^ by proteins proceeds for a long time (>1 h) [3], eventually all ABTS^●^ may be reduced and the purple color becomes evident. The aim of this study was a description of this phenomenon and an explanation of the mechanism of color formation.

## 2. Results

**Figure 1:**
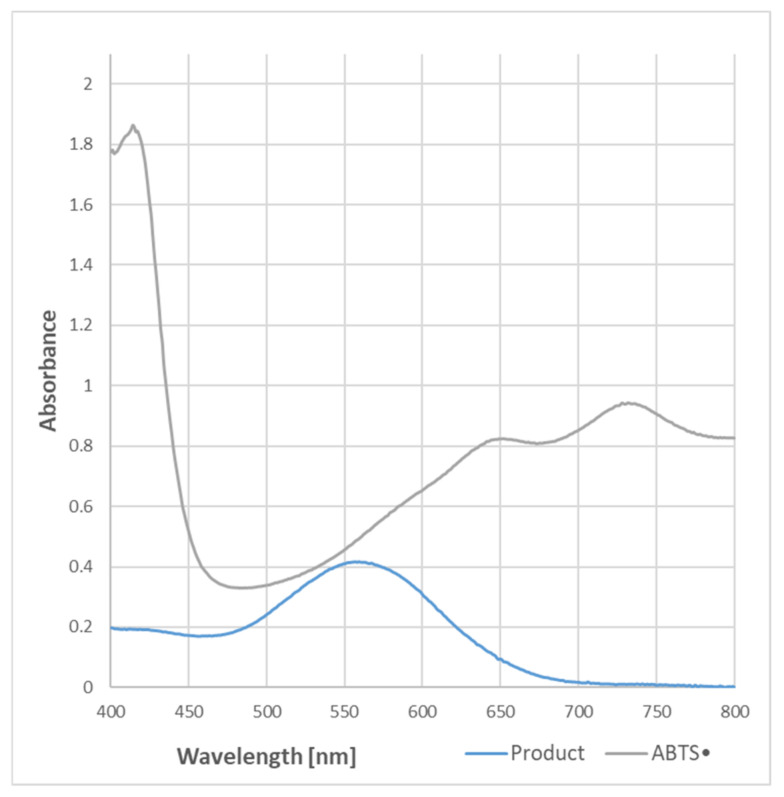
Spectra of ABTS^●^ and the purple product of the reaction of ABTS^●^ (107 μM) with BSA (750 μg/mL) in PBS. Reaction time: 2 h.

The purple color of the samples containing BSA and ABTS^●^ became evident when the protein content was equal to or higher than needed for the total reduction of the ABTS^●^ present in the sample. The absorption spectrum of the purple product of the reaction was completely different from that of ABTS^●^, and had a maximum of 550–560 nm (Figure 1).

**Figure 2:**
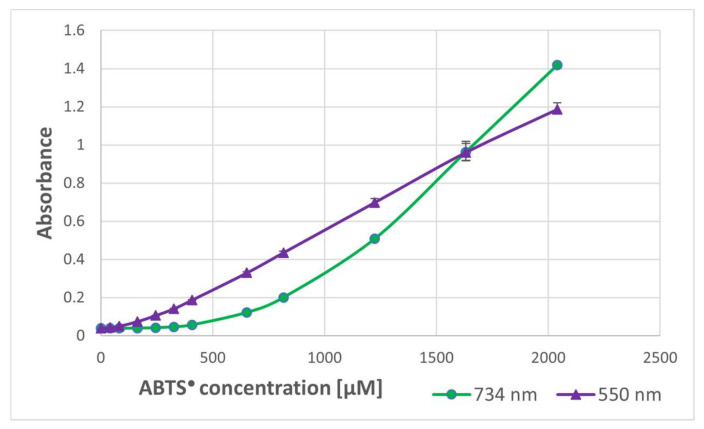
Dependence of the absorbance of the purple product (550 nm) of BSA and ABTS^●^ on the (final) concentration of ABTS^●^. BSA: 10 mg/mL of PBS. Reaction time: 1 h.

In order to check whether the formation of the purple product occurs in parallel to the ABTS^●^ reduction, the dependence of the purple product formation on the amount of ABTS^●^ was followed. As ABTS^●^ also absorbs at 550 nm (the ratio of absorbance at 550 nm and 734 nm was 0.468), the contribution of ABTS^●^ absorbance was subtracted. The dependence of the absorbance of ABTS^●^ and the corrected absorbance of the purple product on the concentration of ABTS^●^ in the sample is shown in Figure 2. These data evidence that the purple product is formed in parallel to the ABTS^●^ reduction, although it does not significantly affect the readings of the assay since it weakly absorbs at the wavelength employed in the assay (734 nm) (Figure 1).

No purple product formation was observed upon the prolonged incubation of BSA with ABTS (non-radical form of the compound, the substrate for the preparation of ABTS^●^).

**Figure 3:**
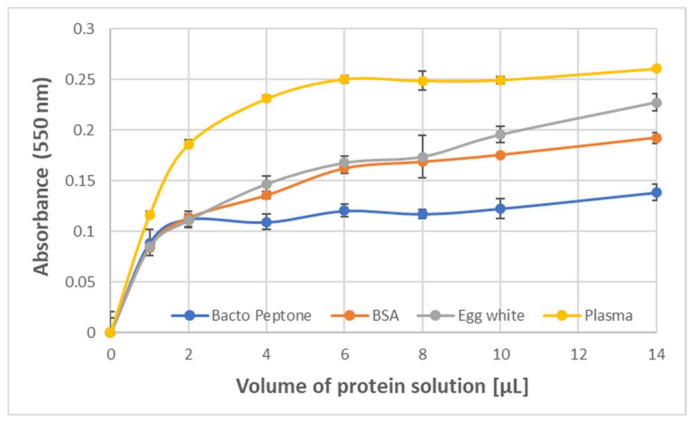
Dependence of the absorbance of the purple product (550 nm) of BSA and ABTS^●^ on the volume of the solution of protein or protein-containing substrate. Bacto Peptone: 20 mg/mL; BSA: 10 mg/mL; egg white (10× diluted): 10.2 mg protein/mL; blood plasma (3× diluted): 20.5 mg protein/mL; ABTS^●^: 1.63 mM in PBS (200 μL). Reaction time: 1 h.

The formation of the purple product was also observed upon the reaction of ABTS^●^ with protein-rich samples such as human blood plasma, egg white, Bacto Peptone, and erythrocyte membranes. The dependence of the amount of the product formed by 1.63 mM ABTS^●^ on the protein concentration showed a saturation starting from about 0.2 mg/mL BSA and egg white protein, and about 0.4 mg/mL Bacto Peptone blood plasma protein (final concentrations) (Figure 3).

**Figure 4:**
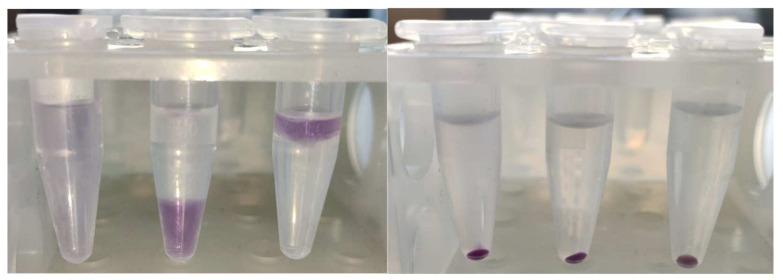
Co-precipitation of the purple product with BSA upon treatment with (from left to right): PBS (no precipitate), diethyl ether, chloroform, ethanol, acetone, and 10% TCA.

The product was firmly bound to BSA; it was co-precipitated with protein by trichloroacetic acid (TCA), ethanol, chloroform, and acetone, leaving a colorless, protein-free layer or supernatant, or lower layer in the case of precipitation with chloroform (Figure 4).

**Figure 5:**
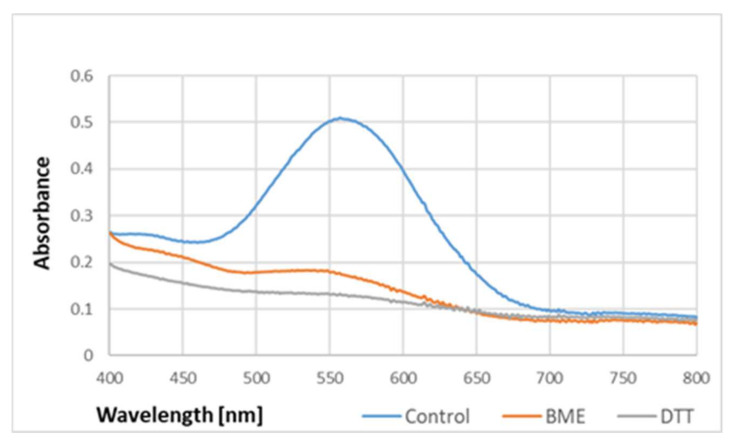
Effect of reducing agents on the absorption spectrum of the purple product of BSA reaction with ABTS^●^. BME, β-mercaptoethanol; DTT, dithiothreitol. The product formed by 10 mg/mL BSA in PBS was added with 5 mM BME or DTT. Reaction time: 1 h.

The absorption spectrum of the purple product was not affected by 2.5% sodium dodecyl sulfate (SDS), but its intensity was diminished by reducing agents such as β-mercaptoethanol and dithiothreitol (Figure 5).

**Figure 6:**
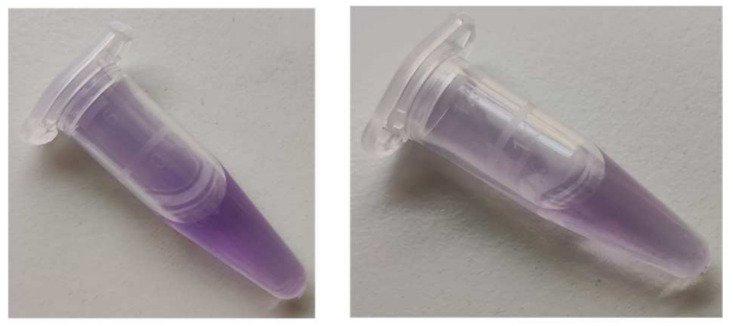
Colors formed by the reaction of BSA (10 mg/mL) with ABTS^●^ (107 μM, final concentrations), left, and by the reaction of tyrosine (2 mM) with ABTS^●^ (107 μM, final concentrations), right. Reaction volume: 1 mL, reaction time: 1 h.

**Figure 7:**
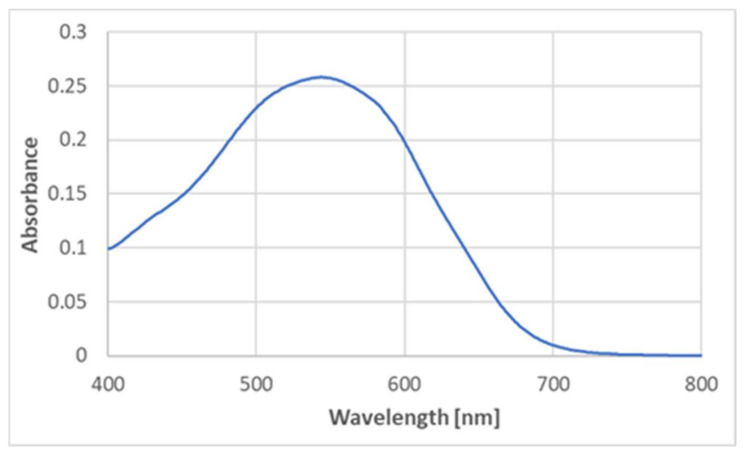
The absorption spectrum of the product of the reaction of ABTS^●^ (125 μM) with tyrosine (2 mM; final concentrations) in PBS. Reaction time: 1 h.

BSA is a protein containing no prosthetic groups, so the product had to be formed by some amino acid constituent(s) of the protein. No product formation was detected upon the reaction of ABTS^●^ with cysteine, cystine, histidine, methionine, serine, leucine, alanine, arginine, and glycine. Glutathione, oxidized glutathione, and glycyl-glycine did not generate the product either, thus excluding the participation of the peptide bond in the product formation. However, tyrosine did react with ABTS^●^, producing a similar color (Figure 6), although the maximum absorption corresponded to slightly lower wavelengths (540–550 nm) (Figure 7).

**Figure 8:**
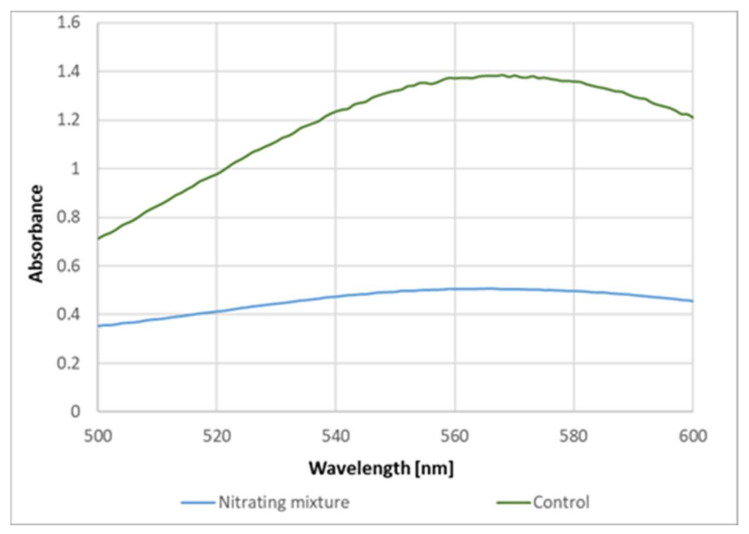
Effect of nitration on BSA on the yield of the purple product.

These data indicate that the formation of the purple product of proteins upon reaction with ABTS^●^ is due to the reaction of protein tyrosyl residues. This conclusion is supported by the inhibition of the purple product formation on the previous nitration of tyrosine residues in BSA (Figure 8).

**Figure 9:**
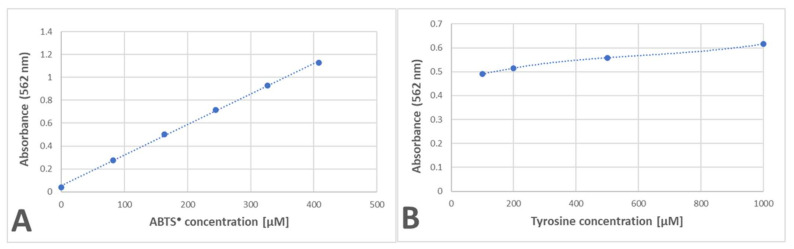
Dependence of the purple product formation of tyrosine on the amount of ABTS^●^ (**A**) and tyrosine (**B**). (**A**): 2 mM tyrosine in 200 μL of PBS added with ABTS^●^ solution; (**B**): 125 μM ABTS^●^ in 200 μL of PBS added with increasing amounts of tyrosine solution. Reaction time: 24 h.

The amount of purple product for the reaction of tyrosine with ABTS^●^ showed a linear dependence on the ABTS^●^ concentration and a much weaker dependence on the tyrosine concentration, with a saturation tendency (Figure 9).

**Figure 10:**
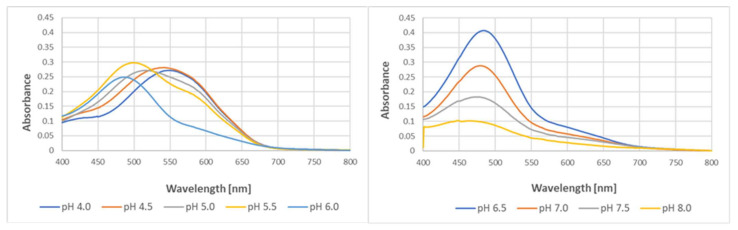
Effect of pH on the formation of the colored product in the reaction of tyrosine (2 mM) with ABTS^●^ (2.5 mM) in 50 mM of acetate of phosphate buffers of various pH levels. Reaction time: 24 h.

pH affected the yield of the reaction and slightly affected the spectra of the purple product of the reaction of tyrosine with ABTS^●^. The optimal pH for the reaction was 6.5, and the yield of the product decreased with a pH decreasing below 6.5 and increasing above this value. The decrease in pH caused a bathochromic shift of the spectra, with the wavelength of maximal absorption increasing from 447 nm at pH 8.0 to 545 nm at pH 4.0 (Figure 10).

**Figure 11:**
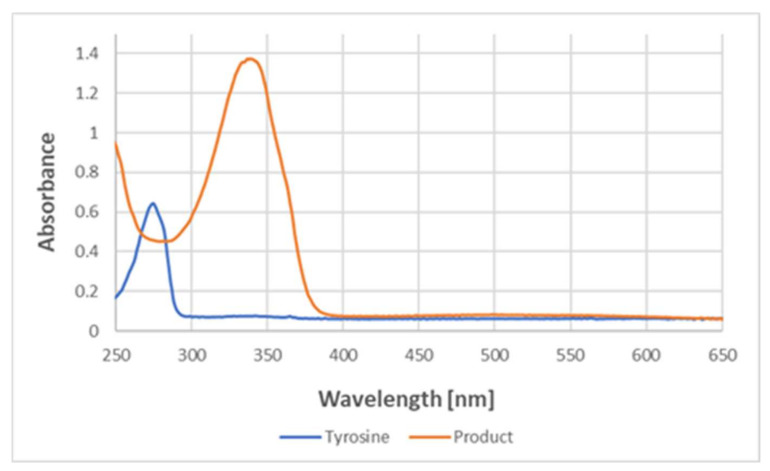
UV–visible spectra of tyrosine and the product of its reaction with ABTS^●^ in PBS. The spectra correspond to the reaction product of 177 μM tyrosine and 147 μM (final concentrations) ABTS^●^.

Absorption spectra in the UV region demonstrated that the reaction with ABTS^●^ led to the disappearance of the absorption of tyrosine at about 280 nm and the appearance of a band at about 340 nm, typical for ABTS (Figure 11).

**Figure 12:**
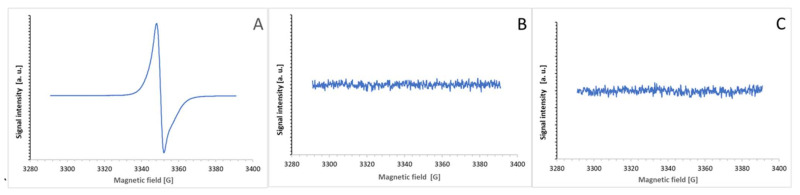
EPR spectra of ABTS^●^ (100 μM, (**A**)) and its reaction products with BSA (20 mg/mL in PBS, (**B**)) and tyrosine (0.4 mg/mL PBS, (**C**)). The reaction products were measured after the total discoloration of ABTS^●^.

The purple products of the ABTS^●^ reaction with BSA and tyrosine were not free radicals and, in contrast to ABTS^●^ (Figure 12A), showed no EPR signal (Figure 12B,C).

Monitoring the spectra of the BSA-ABTS^●^ mixture in time showed that there were no isosbestic points between the spectra of ABTS^●^ and of the adduct (Supplementary Video S1)), which is obvious as the main product (ABTS) does not absorb in the visible range. Thus, the adduct cannot be the only product of the reaction of ABTS^●^ with BSA, apart from the reduction product (ABTS). One of the other byproducts of the reaction is apparently dityrosine.

**Figure 13:**
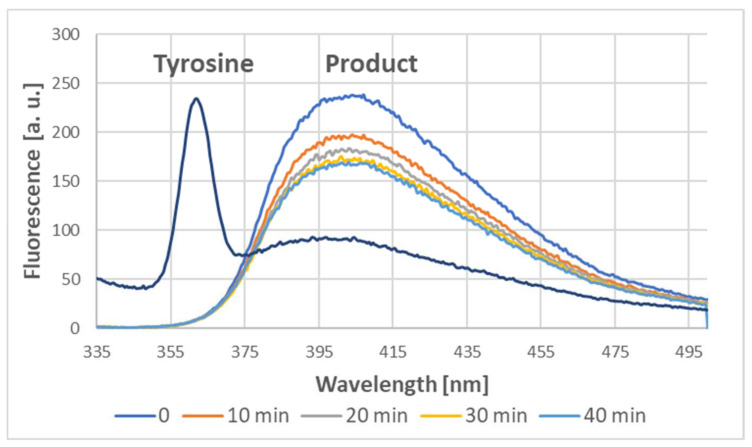
Emission spectra of tyrosine (1 mM) and the product of the reaction between tyrosine (1 mM) and ABTS^●^ (1.63 mM; final concentrations) at various times after the addition of ABTS^●^.

Indeed, the addition of ABTS^●^ to the tyrosine solution also induced dityrosine formation, as demonstrated by the characteristic fluorescence of the product, with a maximum of about 400 nm when excited at 320 nm [4,9,10]. Dityrosine was formed immediately; subsequently, its content somewhat decreased in time (Figure 13).

## 3. Discussion

This study is devoted to the description of the formation and properties of a byproduct of the reaction of proteins with ABTS^●^. To the best of our knowledge, this byproduct was not identified previously in the reaction of ABTS^●^ decolorization. Our results show that the formation of this product is common for various samples containing proteins as components.

The presented results indicate that the reaction of ABTS^●^ with BSA and other proteins, leading to the generation of a purple product, is due to the reaction of ABTS^●^ with protein tyrosine residues. The premises for such conclusions are: (i) a product with a similar spectrum is formed upon the reaction of ABTS^●^ with tyrosine, but not any other amino acid tested, (ii) protein nitration, affecting mainly tyrosine residues [11,12], attenuates the formation of the purple product of BSA. The product was stable on the time scale of hours to days. The purple product of the reaction of ABTS^●^ with tyrosine had an absorption maximum at about 549 nm; the spectrum showed a bathochromic shift with decreasing pH. The spectral difference between the product of tyrosine and BSA reaction with ABTS^●^ is apparently due to the effect of the amino acid environment on the absorption spectra of tyrosine residues in the protein.

The local environment of tyrosine residues in proteins may differ considerably, as demonstrated by differences in their pK_a_ values. The pK_a_ value of free tyrosine is 10.07 (www.peptideweb.com, accessed on 12 May 2023); the pK_a_ value of tyrosine residues in BSA was estimated to be 11.95 [13]. The pK_a_ values of tyrosyl residues of the photoactive yellow protein of *Halobacterium halobium* differ, depending on whether the phenolic side chain is solvent-exposed, buried, or hydrogen-bonded. Solvent-exposed Y76 and Y98 have pK_a_ values of 10.2–10.3. Y118, partly buried within the protein interior, but with the hydroxyl group is not involved in the hydrogen bonding, has pK_a_ values of 11.4–12.0. Hydrogen-bonded and partially buried Y42 and Y94 residues have pK_a_ > 13 [14]. The pK_a_ of tyrosine of human serum apoferritin is unusually low (7.57) but increases up to more than 9.0 upon the addition of sulfate [15]. Thus, there are reasons to expect differences in the reactivity between free tyrosine and tyrosine residues proteins, as well as between individual tyrosine residues in various proteins with ABTS^●^, and differences in the spectral characteristics of the adducts.

The lack of an EPR signal of the product demonstrates that the product is not a free radical. It excludes the possibility of the purple color being due to the presence of the ABTS biradical, possessing a broad absorption spectrum centered at about 540 nm. Moreover, the absorption spectra of the product in the UV range showed a disappearance of the absorption band at about 280 nm, characteristic of tyrosine, and an appearance of the absorption band at about 340 nm, characteristic of ABTS but not ABTS^●^ (the latter has an absorption band at about 420 nm) [8,16], which confirms the non-radical character of the bound ABTS residue. The co-precipitation of the reaction product with BSA indicates its covalent binding to the protein. The most feasible explanation is that the product is an adduct of ABTS to tyrosine.

The probable mechanism of the adduct formation is proposed below.

During the reaction of the ABTS^●^ reduction by a protein or free tyrosine, one-electron oxidation of tyrosine by ABTS^●^ forms the tyrosyl radical:

Tyr-OH + ABTS^●^ → Tyr-O^●^ + ABTS (1)

The main reaction of the so-formed tyrosyl radical is the reduction of another ABTS^●^ radical:

Tyr-O^●^ + ABTS^●^ → Tyr = O + ABTS (2)

where Tyr = O is the quinone product of two-electron tyrosine (Tyr-OH) oxidation.

However, a fraction of the tyrosyl radicals can be engaged in other reactions such as covalent adduct formation (3) and the formation of dityrosine (4) [17]:

Tyr-O^●^ + ABTS^●^ → TyrOH-ABTS (3)

**Figure 14:**
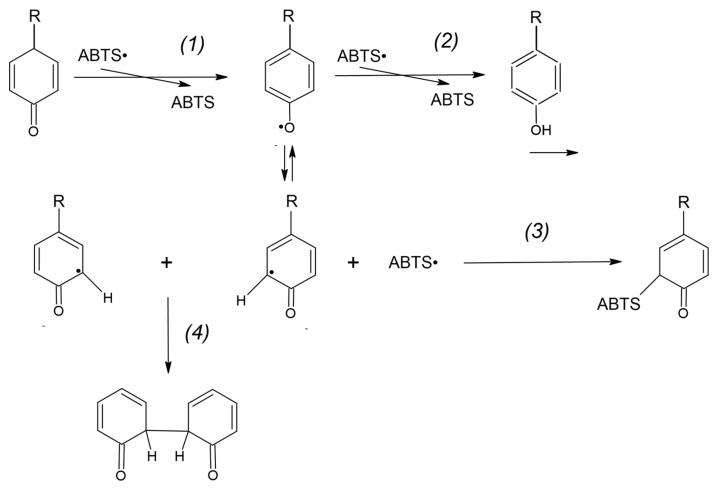
Reactions of tyrosine radicals. Tyrosine radical is formed in the reaction of ABTS^●^ reduction (1) and mainly reacts with the next ABTS^●^ radical (2). It can also form an adduct with an ABTS^●^ radical (3) or dimerize to form dityrosine (4).

Tyr-O^●^ + Tyr-O^●^ → Dityrosine (4)

as presented in Figure 14. Other reactions may also proceed in this system.

There are literature data pointing to the formation of stable tyrosine-ABTS^●^ adducts due to reactions of the tyrosyl radical. The blue laccase of *Sclerotinia sclerotiorum* was rapidly converted to a deep purple form upon turning over ABTS or by the addition of chemically pre-formed ABTS^●^. The color disappeared gradually after ~10 min, presumably decomposed via redox cycling. Differences in the spectra of the laccase-ABTS^●^ adduct (λ_max_ = 575 nm) and for the tyrosine-ABTS adduct (λ_max_ = 555 nm) of ABTS^●^ and free tyrosine formed in a reaction catalyzed by laccase were ascribed to the influence of the protein environment [18]. The reaction of ABTS with lipocalin α1-microglobulin, also leading to the formation of a purple product, conditioned by the formation of ABTS-adducts to at least two tyrosine residues in the protein molecule, was also reported [19].

The formation of covalent adducts between ABTS^●^ and the oxidation products of polyphenols such as phloroglucinol and catechin has been demonstrated. The primary adducts were unstable and formed secondary adducts, releasing part of the ABTS molecule [20], and were identified among the products of ABTS^●^ reactions with polyphenols [21].

Analysis of the purple tyrosine-ABTS adduct led to the conclusion that the adduct was formed in the ortho position of tyrosine, via a bond between nitrogen in the fragment of the ABTS molecule and carbon in the ortho position with respect to the hydroxyl group of tyrosine [19].

The detection of the formation of the purple adduct of lipocalin α1-microglobulin has been interpreted as an argument that this protein acts as a “radical sink” via its radical reductase and scavenging activities. The present results demonstrate that the formation of purple adducts is a general feature of proteins. Most proteins have tyrosine residues, and all proteins are able to reduce ABTS^●^ due to the presence of cysteine, tyrosine, tryptophan, histidine, or cystine residues [22]. Tyrosine residues are one of the amino acid residues most susceptible to oxidative modifications, including one-electron oxidations [23]. Tyrosine radicals, if formed in proteins, are able to form adducts with various radicals [18]. Tyrosine nitration proceeds via the formation of tyrosyl radicals and the subsequent reaction of these radicals with ^●^NO_2_. Several oxidants, such as ^•^OH, ^•^NO_2_, CO_3_^•−^, peroxyl (LOO^•^) and alkoxyl (LO^•^) radicals, oxo-metal compounds such as O = Mn(IV), and Compounds I and II of heme peroxidases, such as myeloperoxidase, can perform the one-electron oxidation of tyrosine in biological systems When Tyr^•^ radical is formed it can have different fates, one of which is the diffusion-controlled reaction with ^•^NO_2_ to form nitrotyrosine [24,25].

The coupling of 3-hydroxyanthranilic acid with protein tyrosine residues, used by some moth species for the tanning of cocoons [26], proceeds through a radical addition of 3-hydroxyanthranilic acid to a tyrosyl radical [27]. Polyunsaturated lipid peroxyl radicals may also give stable peroxide coupling products exclusively at the para position of the tyrosyl phenoxy radicals; the formation of such adducts may terminate lipid peroxidation reactions [28]. The reaction of protein tyrosyl radicals with superoxide produces hydroperoxide, which is reduced to monoxide, yielding a glutathionylated adduct via Michael addition [29]. The formation of adducts between protein tyrosyl radicals and the spin trap 5,5-dimethyl-1-pyrroline N-oxide (DMPO) is the basis of the ingenious immunochemical method of detection of the formation of these radicals using anti-DMPO antibodies [30,31]. As the adduct formed by tyrosyl radicals with ABTS^●^ described in this study has a purple color and its formation can be easily followed spectrophotometrically, it may be a useful indicator of such addition reactions of tyrosine radicals in proteins and peptides.

The present results demonstrate that the adduct formation, as well as dityrosine formation, contribute to the complexity of the apparently simple reaction of ABTS^●^ decolorization. van Overveld et al. found the antioxidant activity of tyrosine in the ABTS^●^ decolorization assay to be 1.48 mol Trolox equivalents (TE)/mol tyrosine after a 5 min reaction [32]. Torkova et al. found a value of 3.38 mol TE/mol tyrosine after a 40.5 min reaction [33]. We obtained an antioxidant activity of 4.07 mol TE/mol tyrosine after a 30 min reaction [22]. In all cases, the antioxidant activity of tyrosine was higher than 1, a value which could be expected if only the phenol group participated in the reaction. Arts et al. demonstrated that the reaction product of the reaction of resorcinol with ABTS^●^ participated in the reaction, causing additional ABTS^●^ reduction [34]. A similar situation may take place in the reaction of tyrosine with ABTS^●^. However, the formation of the tyrosine-ABTS adduct and dityrosine may also contribute to the higher-than-expected antioxidant activity of tyrosine in the reaction of ABTS^●^ decolorization.

## 4. Materials and Methods

### 4.1. Reagents and Disposables

L-Arginine (CAS no. 74-79-3, cat. no. 11009, purity ≥ 99.5%), L-methionine (CAS no. 63-68-3, cat. no. M9625, purity ≥ 98%), L-serine (CAS no. 56-45-1, cat. no. S4500, purity ≥ 99%), L-leucine (CAS no. 61-90-5, cat. no. L8000, purity ≥ 98%), L-lysine monohydrochloride (CAS no. 657-27-2, cat. no. L5626, purity ≥ 98%), β-Alanine (CAS no. 107-95-9, cat. no. 146064, purity ≥ 99.5%), sodium dodecyl sulfate (SDS) (CAS no. 151-21-3, cat. no. L4509, purity ≥ 98.5%), glycine (CAS no. 56-40-6, cat. no. 410225, purity ≥ 98.5%), cysteine (CAS no. 52-90-4, cat. no. 168149, purity ≥ 97%), L-glutathione reduced (CAS no. 70-18-8, cat. no. G4251, purity ≥ 98%), L-glutathione oxidized (CAS no. 27025-41-8, cat. no. G4376, purity ≥ 98%), glycyl-glycine (Gly-Gly) (CAS no. 556-5-3, cat. no. G1002, purity ≥ 99%), sodium nitrite (CAS no. 7632-00-0, cat. no. 8222850100, purity > 98%), and citric acid monohydrate (CAS no. 5949-29-1, cat. no. 1002441000, ACS reagent) were purchased from Merck (Poznań). L-Cystine (CAS no. 56-89-3; cat. no. 2/03/75 purity ≥ 98%) was obtained from Biomed (Lublin, Poland). DL-Dithiothreitol (DTT) (CAS no. 3483-12-3; cat. no. DTT001.5, purity ≥ 99.5%), L-histidine (CAS no. 71-00-1; cat. no. HIS100.25, purity ≥ 98.5%), phosphate-buffered saline (PBS; cat. no. PBS404.200), sodium phosphate monobasic (CAS no. 10049-21-5; cat. no. SPM306.500, purity 98–103%), and sodium phosphate dibasic (CAS no. 7782-85-6; cat. no. SPD579.1, purity 98–102%) came from LAB EMPIRE (Rzeszow, Poland).

Tryptophan (CAS no. 73-22-3, cat. no. 4858, purity ≥ 98.5%) and tyrosine (CAS no. 60-18-4, cat. no. T207, purity ≥ 99%) were purchased from Roth (Zielona Góra, Poland). 2,2′-Azino-bis (3-ethylbenzthiazoline-6-sulfonic acid) (ABTS) (CAS no. 504-14-6, cat. no. 10102946001, purity ≥ 99%) came from Roche (Warsaw, Poland).

Ethanol (CAS no. 64-17-5, cat. no. 396480111, purity ≥ 99,8%) and sodium acetate anhydrous (CAS no. 127-09-3, cat. no. BN60/6191, purity ≥ 99%) were from Avantor Performance Materials Poland (Gliwice, Poland). Acetic acid (CAS no. 64-19-7, cat. no. 425687339, purity 80%), hydrochloric acid (CAS no. 7647-01-0, cat. no. 115752837, 35–38%), hydrogen peroxide (CAS no. 7722-84-1, cat. no. 118851934, 30%), sodium nitrite (CAS no. 7632-00-0, cat. no. 792690115, purity ≥ 97.5%), chloroform (CAS no. 67-66-3, cat. no. 112344305, purity ≥ 98.5%), acetone (CAS no. 67-64-1, cat. no. 111024800, purity ≥ 99.5%), and diethyl ether (CAS no. 60-29-7, cat. No. 113842106, purity ≥ 99.5%) were provided by Chempur (Piekary Śląskie, Poland).

Albumin Fraction V (BSA) (CAS no. 9038-46-8, cat. No. A1391,0025, purity ≥ 97%) was bought from AppliChem (Darmstadt, Germany), β-mercaptoethanol (BME) (CAS no. 60-24-2, cat. No. Z523A, 48,7%) were from Promega (Madison, WI, USA), and sodium hydroxide (CAS no. 1310-73-2, cat. no. 056992, purity ≥ 98%) came from Warchem (Warsaw, Poland). Bacto Peptone (cat. no. 211677) was from Becton and Dickinson Company (Le Pont de Claix, France). Distilled water was purified using a Milli-Q system (Millipore, Bedford, MA, USA). Transparent flat-bottom 96-well plates (cat. no. 655101) (Greiner, Kremsmünster, Austria) were used for the assays. Absorptiometric measurements were performed in a Spark multimode plate reader (Tecan Group Ltd., Männedorf, Switzerland).

Stock solutions of BSA were made in PBS. Chicken egg white was diluted 10 times with PBS. Human erythrocyte membranes were prepared according to a modification [35] of the method of Dodge et al. [36].

### 4.2. ABTS^●^ Decolorization Assay

The assay was carried out as described previously [3]. In a standard assay, aliquots of amino acid or protein solution containing increasing amounts of the protein causing a reduction of not more than 90% of the ABTS^●^ present in the sample were added to wells of a 96-well plate containing 200 μL of solution diluted so as to provide absorbance of 1.0 at 734 nm in a plate reader. The decrease in absorbance was read after 30 min at ambient temperature. In the experiments reported here, greater amounts of protein or higher concentrations were also used, and longer incubation times were employed as indicated in figure legends.

### 4.3. Nitration of Tyrosine Residues in BSA

BSA (20 mg/mL in 0.2 M sodium citrate buffer, pH 5, 100 μL) was added with 20 μL of 200 mM sodium nitrite and 20 μL of 200 mM H_2_O_2_, mixed immediately, and incubated at room temperature for 2 h. The same amounts of sodium nitrite and hydrogen peroxide were added twice more in 2 h intervals.

### 4.4. Electron Spin Resonance Measurements

Electron paramagnetic resonance (EPR) measurements were performed on a Bruker multifrequency and multi-resonance FT-EPR ELEXSYS E580 spectrometer (Bruker Analytische Messtechnik, Rheinstetten, Germany) operating at the X-band (9.378989 GHz). The following settings were used: modulation amplitude, 0.4 G; modulation frequency, 100 kHz; microwave power, 94.64 mW; power attenuation, 2 dB; conversion time, 25 ms; sweep time, 102.4 s; powder sample: central field, 3501 G; scan range, 7000 G; liquid sample: central field, 3353.15 G; scan range, 100 G, accumulate, 10. The spectra were recorded and analyzed using Xepr 2.6b.74 software.

### 4.5. Fluorimetric Assessment of Dityrosine Formation

Tyrosine (2 mM) in 50 mM phosphate buffer, pH 8.0, was added with an equal amount of 1.63 mM ABTS^●^ solution. Fluorescence spectra were measured immediately and at 10 min intervals in a Hitachi F-2500 spectrofluorimeter (Tokyo, Japan) at an excitation wavelength of 320 nm [9,10], using excitation and emission slits of 5 nm.

## Data Availability

Data available on reasonable request from the corresponding author.

## Supplementary Materials

The following supporting information can be downloaded at: https://www.mdpi.com/article/10.3390/ijms24108912/s1.
